# Substantial Intra-Individual Variability in Post-Prandial Time to Peak in Controlled and Free-Living Conditions in Children with Type 1 Diabetes

**DOI:** 10.3390/nu13114154

**Published:** 2021-11-19

**Authors:** Emily Bell, Sabrina Binkowski, Elaine Sanderson, Barbara Keating, Grant Smith, Amelia J. Harray, Elizabeth A. Davis

**Affiliations:** 1 Children’s Diabetes Centre, Telethon Kids Institute, University of Western Australia, Perth 6009, Australia; Emily.Bell@telethonkids.org.au (E.B.); Elaine.Sanderson@health.wa.gov.au (E.S.); Grant.Smith@telethonkids.org.au (G.S.); Amelia.Harray@telethonkids.org.au (A.J.H.); Elizabeth.Davis@health.wa.gov.au (E.A.D.); 2 Department of Endocrinology and Diabetes, Perth Children’s Hospital, Perth 6009, Australia; Barbara.Keating@health.wa.gov.au; 3 School of Population Health, Curtin University, Bentley, Perth 6009, Australia; 4 Division of Paediatrics, School of Medicine, University of Western Australia, Perth 6009, Australia

**Keywords:** type 1 diabetes, paediatric diabetes, glycaemic control, CGM, variability, time to peak

## Abstract

The optimal time to bolus insulin for meals is challenging for children and adolescents with type 1 diabetes (T1D). Current guidelines to control glucose excursions do not account for individual differences in glycaemic responses to meals. This study aimed to examine the within- and between-person variability in time to peak (TTP) glycaemic responses after consuming meals under controlled and free-living conditions. Participants aged 8–15 years with T1D ≥ 1 year and using a continuous glucose monitor (CGM) were recruited. Participants consumed a standardised breakfast for six controlled days and maintained their usual daily routine for 14 free-living days. CGM traces were collected after eating. Linear mixed models were used to identify within- and between-person variability in the TTP after each of the controlled breakfasts, free-living breakfasts (FLB), and free-living dinners (FLD) conditions. Thirty participants completed the study (16 females; mean age and standard deviation (SD) 10.5 (1.9)). The TTP variability was greater within a person than the variability between people for all three meal types (between-person vs. within-person SD; controlled breakfast 18.5 vs. 38.9 min; FLB 14.1 vs. 49.6 min; FLD 5.7 vs. 64.5 min). For the first time, the study showed that within-person variability in TTP glycaemic responses is even greater than between-person variability.

## 1. Introduction

Postprandial hyperglycaemia is a major contributor to overall glycaemic control and glycaemic variability [1]. Minimising the time spent above the target blood glucose range has been shown to reduce the risk of long-term micro and macrovascular complications associated with type 1 diabetes (T1D) [2,3]. Achieving optimal post-prandial glucose control requires matching not only the quantity but also the timing of insulin delivery to the appearance of glucose in the bloodstream after a meal. Delivery of meal insulin bolus to achieve optimal glucose levels in the recommended target range (3.9–10 mmol/L) remains a challenge in T1D management for children and adolescents [4,5]. Even with the growing uptake of continuous glucose monitoring (CGM) and the closed loop system, an optimal strategy has still to be identified [6].

Current international guidelines to control postprandial glucose excursions recommend administering insulin 15 to 20 min before eating [7]. The dose of insulin is calculated using the quantity of carbohydrates in the meal, the glucose level before the meal, and the person’s individualised carbohydrate ratio (ICR) and insulin sensitivity factor (ISF) [4]. Accurate carbohydrate counting is an essential skill for determining mealtime insulin boluses, however, its impact on improving glycaemic outcomes and reducing post-prandial hyperglycaemia have been modest [8,9,10]. In addition to estimating the carbohydrate content of a meal, evidence suggests fat and protein content should also be considered in insulin dosing algorithms [11,12,13,14].

Recently, Keating and colleagues [15] used an insulin clamp study design to precisely measure the amount and timing of insulin required to maintain euglycemia for meals of different macronutrient compositions. Through an examination of the time to peak (TTP) glycaemic response and area under the curve (AUC), this study found significant differences between participants in the timing and dose of insulin requirements, yet it also found similarities within the same person, even after consuming meals of different macronutrient composition [15]. However, this study was unable to comprehensibly assess within-person variation as each different meal was only consumed on one occasion. Nonetheless, this study suggests that a ‘one-size-fits-all’ approach to insulin dosing may not be appropriate. The study also raises the question of whether children display similarities in their TTP glycaemic response, regardless of the macronutrient composition of meals. There is currently limited understanding of the daily variability in the TTP glycaemic response of children with T1D in a free-living, uncontrolled environment.

To minimise postprandial hyperglycaemia, the timing of insulin delivery prior to a meal should be tailored to the TTP glycaemic response. If consistency within an individual in the timing of glucose response to specific meals can be identified, this will support the development of a more personalised approach to insulin dosing advice as opposed to relying on macronutrient composition alone.

Previous studies in adults with T1D and type 2 diabetes T2D have identified between-person and within-person variation in the TTP glycaemic response under free-living conditions, however, there are limited studies examining this variation in children or adolescents with T1D [16,17,18].

The present study aimed to examine the within-person and between-person variability and consistency in TTP glycaemic response after consuming breakfast under controlled conditions and breakfast and dinner meals under free-living conditions. The purpose was to investigate whether intra-individual similarities of postprandial excursions exist under controlled conditions and can be identified in an uncontrolled environment to provide guidance in personalising advice on the timing of pre-prandial insulin dosing.

## 2. Materials and Methods

### 2.1. Participants

This 5-week prospective observational study recruited participants who met the following inclusion criteria: (1) aged 8 to 15 years; (2) duration of T1D ≥ 1 year; (3) most recent haemoglobin A1c (HbA1c) ≤ 9%; (4) using CGM; (5) on multiple daily injections (MDI) or an insulin pump for >6 months. Exclusion criteria included: (1) diseases affecting digestion and absorption of nutrients, including coeliac disease; (2) those with an allergy or intolerance to the study breakfast meal.

### 2.2. Study Design

Once eligibility was established and consent provided, participants visited Perth Children’s Hospital on one occasion for a baseline visit. This was followed by a two-week run-in period to optimise insulin dosing, a six-day controlled period where participants consumed a provided standard breakfast each morning, and then a 14-day free-living period. Participants’ CGM data were collected from 30 min before to 3 h after the six controlled breakfast meals and the 14 free-living breakfasts and dinners. Potential participants were identified from the Western Australian Children’s Diabetes Database and invited to participate in the study via email or phone.

### 2.3. Baseline Assessment

Participants attended one initial visit at Perth Children’s Hospital (PCH) with the research team. Baseline data including height, weight, age, and insulin regimes were collected. Most recent HbA1c was collected after consent from the Western Australian Children’s Diabetes Database. Participants were provided with a study resource pack and their breakfast meals for the six-day controlled study period, as outlined in Table 1. Participants were instructed to calibrate their CGM with capillary blood glucose readings twice a day as per device recommendations to maximise sensor accuracy.

### 2.4. Run-In Period

Two weeks prior to commencing the study period, participants were contacted by the study doctor to review their blood glucose levels (BGL). Adjustments to participant’s insulin regimen, ICR, and ISF were made, if necessary, to increase the likelihood of the participants’ BGL being between 4 and 8 mmol/L prior to consuming the standard breakfast during the controlled study period.

### 2.5. Controlled-Living Period

Participants consumed a standard study breakfast containing 43.6 g of carbohydrate for six consecutive days. This consisted of foods commonly eaten by Australian children: cereal, milk, and toast. Meal composition is displayed in Table 1. The relatively high carbohydrate content of this meal was intended to dilute the effects of other macronutrients, such as fat or protein, which can prolong the postprandial glycaemic response [19]. Participants who could not eat the entire meal were asked to record the quantity they consumed on the first study day and keep this consistent for the remainder of the controlled study days.

If blood glucose was <4 mmol/L or >8 mmol/L prior to commencing the standard breakfast during the controlled period, participants were instructed to skip this day and continue with the study on the next day. On each study day, participants were instructed to give insulin 15 min before beginning the standard breakfast, using their individualised ICR and the carbohydrate content of the meal to calculate their insulin bolus.

To minimise confounding factors, participants were asked to give insulin in the same site, to eat breakfast in the same 60-min interval, and to finish the standard breakfast within 20 min. Participants were asked to avoid vigorous physical activity, food, drinks (other than water), and extra bolus insulin in the three-hour postprandial period. Participants returned to their normal daily routines following this period.

If sensor glucose fell below 4 mmol/L or symptomatic hypoglycaemia occurred, participants were instructed to confirm this through a capillary blood glucose measurement. If this measurement was less than 4 mmol/L, participants followed their usual management for hypoglycaemia treatment and the study day was terminated.

Participants were asked to record information in a log-book to aid in data analysis and confirm protocol adherence. A member of the research team was in regular contact with participants via phone to obtain information about adverse events and support the study protocol to be followed. Log-books were reviewed upon completion for each study period in order to confirm participant adherence to the study protocol and to determine participants eligible for analysis and those with insufficient data. Collected log-books were inspected for the complete records of time of insulin given (control and free-living period), start time of meal (control and free-living period) and meal finish time (control period).

### 2.6. Free-Living Period

Participants returned to their regular daily routines for 14 days and kept a log-book for breakfast and dinner to capture data points reflective of two different physiological stages, which included time of insulin bolus, direction of glucose trend arrow prior to eating, and meal start time. Participants were not required to record the macronutrient composition of their meals during this period to minimise participant burden and the potential for reactivity and social desirability bias; this study did not request for participants to record their dietary intake throughout the free-living period. 

### 2.7. Measures

Baseline demographics of height, weight, and glycaemic control were collected. During the controlled period, only post-breakfast data was collected, as this early morning time point reflects a fasting state [20] and thus minimises potential cofounding factors, such as previous meals and daily physical activities.

Blood glucose before meals was defined as the capillary blood glucose reading before breakfast in the controlled condition. During the free-living breakfast (FLB) and free-living dinner (FLD) periods, blood glucose before each meal was defined as the closest sensor blood glucose to the meal start time (within 20 min).

Participants used their personal CGM device (DexCom, Inc., San Diego, CA, USA or Medtronic, Minneapolis, MN, USA) during the study period. Sensor glucose readings were measured every five minutes. CGM data was uploaded to the online proprietary software Diasend®(Glooko AB, Göteborg, Sweden) or CareLink™ (Medtronic, Northridge, CA, USA) by participants. R, version 3.6.1 (R-Foundation, Boston, MA, USA), was used via R Studio, version 1.2.1335 (R Studio, Boston, MA, USA), to extract CGM data for the three-hour postprandial period after each meal for analysis. This post-meal time frame was based on previous research demonstrating that TTP glucose excursion after breakfast is usually reached within 90 min [18]. Study days were excluded if a substantial CGM dropout of more than 20 consecutive minutes was observed in the three-hour period.

For each meal, TTP was defined as the time taken to reach the highest recorded glucose value in the three hours after eating [17,18,21,22]. Participants with less than three TTP recordings during the controlled, FLB, or FLD period were excluded.

### 2.8. Statistical Analyses

The sample size was calculated based on the clinically acceptable precision (30%) of the upper limit of the confidence interval (CI) around the inter-individual standard deviation. For the upper limit of the 95% CI to be within 30% of the estimated standard deviation, a sample size of 37 was required. To allow a drop-out rate of 20%, 47 participants were to be recruited.

A participant’s mean TTP across study days for the controlled breakfast (CB), FLB, and FLD condition was calculated. Using these values, the overall mean and SD for each condition was calculated (person-averaged TTP). A series of paired *t*-tests were then used to compare the person-averaged TTP across the three conditions (CB, FLB, and FLD).

For each of the three conditions (CB, FLB, and FLD), a separate unconditional linear mixed model was conducted with TTP as the dependent variable, including a fixed intercept and random intercept (for participant); the variance in TTP was broken down into intra-individual and inter-individual components and presented as standard deviations with 95% confidence intervals. The intraclass correlation coefficient (ICC) was also calculated via the mixed model.

To examine potential predictors of TTP glucose response, the mixed model for each study condition was expanded to include the following covariates: age, body mass index (BMI) z-score, sex, HbA1c, and blood glucose before meal. For the free-living condition models, the pre-bolus time was also included. All statistical analyses were performed using Jamovi, version 1.0.5.0 (Jamovi, Sydney, Australia). Figures were created using R, version 3.6.1 (R Foundation, Boston, MA, USA) via R Studio, version 1.2.1335 (R Studio, Boston, MA, USA). Results were considered significant if *p* < 0.05.

## 3. Results

A total of 30 participants completed the study. Upon log-book review, four were excluded from the controlled period data set due to protocol breaches and missing CGM data (valid *n* = 26). Four additional participants were removed from the inter-individual variation analysis, due to eating a partial amount of the standard breakfast each day or for substituting breakfast items (valid *n* = 22). All 30 participants successfully completed the free-living period. One participant was removed from the FLD dataset due to insufficient data (valid *n* = 29). During the controlled period, a total of 22 hypoglycaemia events in 14 participants were recorded. Of these, 11 events occurred during the three-hour post-breakfast period, resulting in the exclusion of these data points. Participant demographics can be seen in Table 2. In total 870 CGM traces (*n* = 126 for CB, *n* = 350 for FLB, and *n* = 394 for FLD) were analysed (Table 3).

### 3.1. Time to Peak Glucose

**Figure 1:**
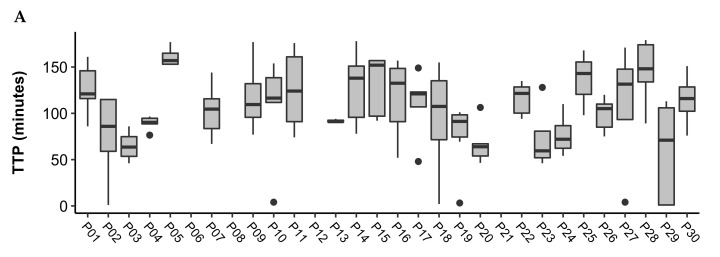

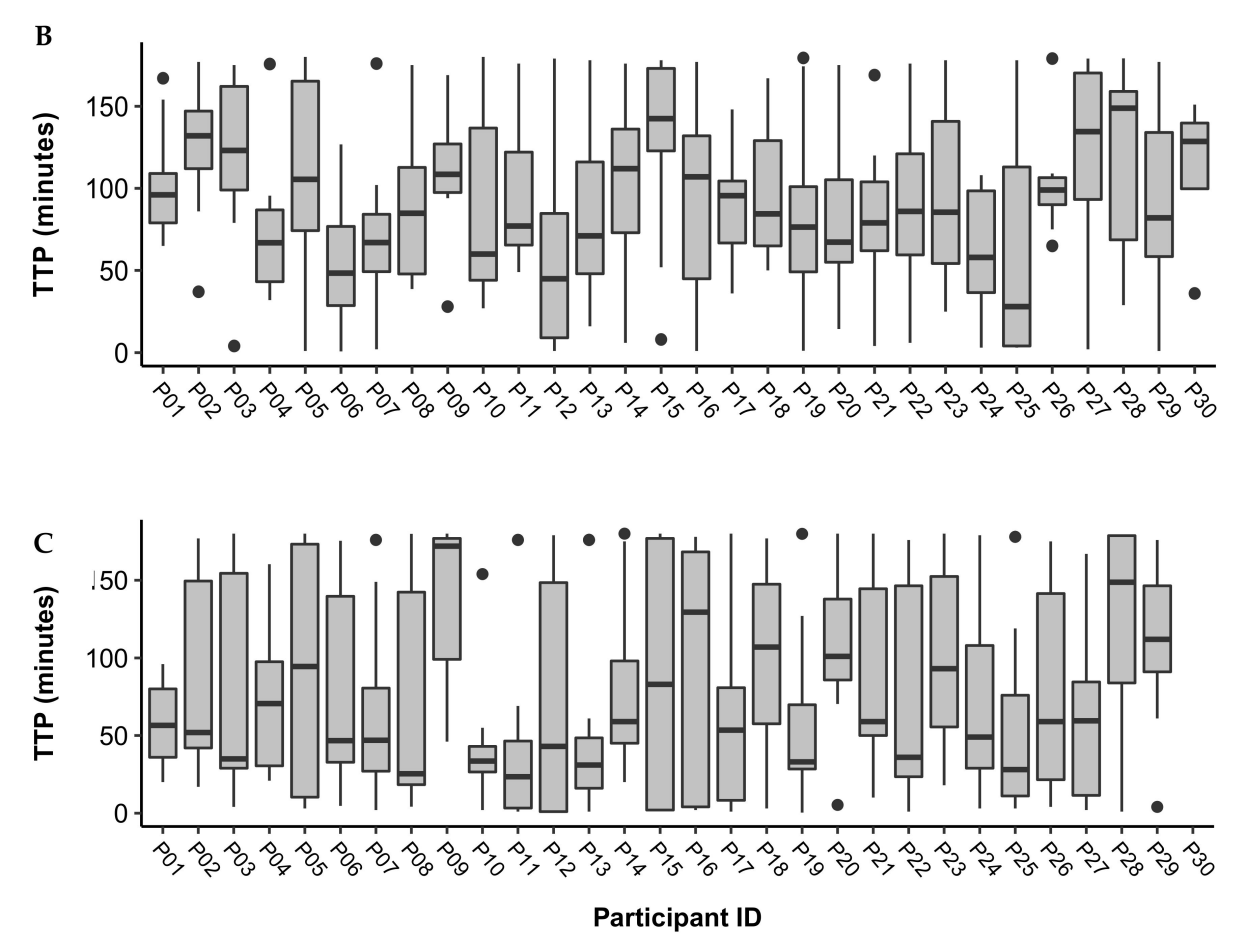
Boxplots of time to peak (TTP) in minutes across study days for participants during the (**A**) Controlled, (**B**) FLB, and (**C**) FLD periods. Outliers are represented by black dots. The mean (SD) study days included in the analysis for each participant was 5 (1) days for the controlled period; 12 (2) days for FLB, and 10 (2) days for FLD.

The person-averaged TTP was as follows for each of the conditions: Controlled = 104.5 (26.6); FLB = 93.2 (19.9); FLD = 79.5 (22.3). The person-averaged TTP for each condition is displayed in Table 3. Figure 1 displays boxplots of the TTP in minutes for each participant across the controlled, FLB, and FLD conditions. The mean (SD) study days included in the analysis for each participant was 5 (1) days for the controlled period; 12 (2) days for FLB, and 10 (2) days for FLD. Pairwise comparisons showed the following differences: Controlled vs. FLB difference = 11.3, *p* = 0.15; Controlled vs. FLD difference = 25.0, *p* = 0.003; FLB vs. FLB difference = 13.7, *p* = 0.007.

### 3.2. Within- and Between-Person Variation

Mixed model outputs of the within- and between-person variation in TTP is depicted in Table 3. The within-person deviation was larger than the between-person standard deviation across all conditions. ICC was less than 0.5 across all conditions.

In the conditional mixed models, no covariates were statistically significantly associated with the TTP glycaemic response in the CB and FLB condition (Table 4). In the FLD conditions, sensor glucose before eating (SG Bef) was the only covariate found to have a significant association with TTP (7.0 min, 95% CI −8.8 to −5.3 min: *p* < 0.001). Estimates of within- and between-person variability in the conditional models were similar to the unconditional models.

## 4. Discussion

The present study challenges the management approach that many clinicians adopt for children living with T1D: that with correct timing and accurate carbohydrate counting, postprandial glycaemia can be consistently in-target. For the first time, this study provides evidence on the within-person and between-person variability in the postprandial glycaemic response for children living with T1D. Under both controlled and free-living conditions, substantial variability was observed within and between individuals in the TTP glycaemic response (Table 3). It was hypothesised that the within-person variability in the TTP would be less than the variability between individuals. Across all conditions, however, the within-person variability in the TTP was found to be greater than the variability between participants. This indicates that within-person fluctuations are responsible for a significant portion of the overall variability. This poses challenges in developing an individualised approach to the timing of insulin delivery, as daily variations within a person must be factored into expectations.

Previous adult studies have identified both within-person and between-person variability in the glycaemic response [16,17,18], however, this study is the first to investigate this variability in a paediatric cohort. Daenen et al. [18] and Johansen et al. [16] concluded that the substantial within-person and between-person variability in the TTP response interferes with the ability to determine an optimal time to measure postprandial blood glucose in adults with T1D or T2D. In addition, Cichosz et al. [17] investigated within-person variability in the TTP response under free-living conditions in adults with T2D and found that TPP was least variable during breakfast, which is consistent with the current study. The ICC identified by Cichosz et al. [17] during breakfast, however, was considerably higher than what was found by the current study (0.6 vs. 0.075), indicating that TTP was more variable in this paediatric study. These differences are most likely due to the demographics of the study population and associated differences in energy requirement and hormonal changes in children versus adults.

A notable finding of the current study is the substantial within-person variation identified in the TTP response. The extent of the within-person variability was unexpected in the controlled period given that various factors, including the meal composition, were controlled. Due to the high within-person variation, no overall similarities in TTP responses in individuals were identified in the controlled and free-living conditions. Clinicians will have heard the frustration with difficulty in achieving reproducible postprandial glucose patterns from families who have children living with diabetes. Despite this, our current guidelines consistently recommend insulin to be given 15-20 mins before a meal. It was expected that under these controlled conditions, the TTP response would remain relatively similar within a person. The high degree of variability between each controlled study day, however, suggests that there are alternate factors influencing the TTP response within an individual, such as fluctuations in psychological and physiological states. Importantly, examination of the variance components contributing towards variability in the TTP response found that the within-person variance was more substantial than the between-person variance under all conditions (Table 3). This suggests that the daily variations within a person may be responsible for a large portion of the variability found in a random population sample at any point in time. This finding has relevance for the interpretation of findings from other studies. It cannot be assumed that variance can be attributed to between-person variability alone. The results of this study also support consideration of within-person variability in clinical practice. Daily fluctuations in glucose response to meals can produce unpredictable hyper- and hypoglycaemia that make the management of diabetes difficult. An increased understanding of the difficulties faced by children with T1D can not only aid clinicians in their education and management strategies, but also encourage clinicians to show increasing empathy towards each family’s experience—where even those who follow the strictest dosing regimens can still experience moments of poor glycaemic control.

The findings of the within-person variability in the TTP glucose response was expected in the free-living condition due to the study design and might be related to different meal sizes and meal compositions. In the controlled period, the high degree of within-person variability may be explained by the nature of an at-home study where environmental, psychological, and physiological stimuli can exert differential effects on BGLs from day to day [23,24,25,26,27,28,29,30]. Acute psychological stress has been shown to delay the decrease in the postprandial excursion in adults with T1D and contribute to chronic hyperglycaemia [23,24]. Studies have also shown that the absorption of insulin may vary within a person, due to the preparation or depth of injection, or the temperature or physical activity levels on a given day [25,26]. Additionally, the age group included in this study were likely to be undergoing puberty, which has been associated with deteriorated glycaemic control due to changes in body mass, behaviour, and the psychosocial environment [27]. Although the current study identified no differences in sex, the menstrual cycle has also been associated with cyclical changes in BGL which may further contribute to the variability in female participants [28,29,30]. These factors may explain why the current study found a substantial amount of within-person variability during the controlled conditions, compared to the study conducted by the Children’s Diabetes Centre, which used a precise insulin clamp technique to measure the timing when insulin was required for different meals [15], highlighting the importance of addressing different stages of puberty and its implication on time to peak glycaemic response in a bigger cohort study. The aforementioned study did not take into account the plethora of environmental, social, and emotional stimuli that can affect BGLs within a person from day to day. Instead, it gave a static representation of an individual’s postprandial response on a particular day. As such, the findings of the current study present an increasingly realistic picture of the TTP response for children living with T1D, particularly highlighting the variation that occurs within a person on a daily basis.

Our study found that in the controlled and free-living breakfast conditions, there were no covariates that could explain the observed within- and between-person variability in the TTP (Table 4). This further highlights the difficulties that children living with T1D face in maintaining their glycaemic control. Purpose-designed studies should be conducted in the future to further examine specific predictors of variability in the TTP. During the FLD period, sensor glucose was the only covariate we examined to have a relationship with the TTP glycaemic response, with a higher sensor glucose reading prior to eating resulting in a shorter TTP. The relationship between the TTP response and sensor glucose level at dinner could be explained by various factors, including daily activities, previous food intake, insulin on board, and differences in pre-prandial insulin dosing causing fluctuations in BGL before dinner. Comparatively, the time period before breakfast is a representation of a fasting state [20], and thus the postprandial glucose response is not influenced by these daily activities or excess insulin, as it is during dinner.

Various physiological mechanisms, such as the microbiome [31,32] and gastric emptying [33,34], have been proposed to explain the between-person variability in the postprandial glycaemic response. The microbiome has recently been identified as an independent predictor of an individual’s postprandial glycaemic response in participants without T1D [31,32]. Gastric emptying has also been shown to exhibit a wide range of inter-individual variability, ranging from 1–4 kcal/min, whilst remaining relatively stable within a person over time [33,34]. This would understandably lead to a variable timing of glucose deposition into the blood for each individual, resulting in a variable TTP between individuals.

A strength of conducting this study in the home environment is that these results are translatable to a real-world setting. This study is also strengthened through its design which includes both controlled and free-living conditions, giving a comprehensive assessment of the degree of within-person and between-person variability in the TTP response. The current study’s use of CGM, as opposed to self-monitoring of blood glucose, also allowed for a more precise method of quantifying BGLs and determining the TTP [6]. Additionally, an important strength of this study was the use of a single researcher to recruit, liaise, and collect data from participants, thus reducing bias.

The limitations of the study include that non-adherence to study design led to some study days being excluded, reducing the number of data points included in the analysis. This may have led to certain daily patterns in TTP being overlooked. However, the average number of datasets for each individual was still considerably large, with 5 days for the CB, 12 days for FLB, and 10 days for FLD conditions. Additionally, the current analysis only examined the TTP responses of 30 individuals, which was less than the 37 participants required. As such, the confidence intervals of the standard deviation are wider than the 30% that was desired. The reduced sample size particularly underpowered the exploratory analysis of predictor variables, and future research is needed in this area to understand the factors contributing to both within- and between-person variability in TTP more comprehensively. A further limitation of the present study is that although the majority of participants (83.3%) used a DexCom G5 device (DexCom, Inc., San Diego, CA, USA), 16.7% of participants used a different CGM device, which may have contributed to variability in TTP between individuals and was not accounted for. In the future, this study could also be strengthened by conducting sub-studies with different meal compositions for different age groups with different stages of puberty and individual daily energy requirements, to increase comparability between participants.

In the modern age of CGM use, families can directly visualise this variation in postprandial glycaemia. Translation of this new knowledge about variability of response to both clinicians and families to set realistic expectations will be an important next step of these findings.

## 5. Conclusions

In conclusion, it is clear that glycaemic responses display both within-person and between-person variability in children living with T1D, with results suggesting within-person variability in the TTP response may be more substantial than the variability between individuals. These findings emphasise the ongoing challenge for children living with T1D and their families in attaining optimal glycaemic control. There is no universal approach to insulin dosing, or to diabetes management, due to the substantial variation that occurs within and between individuals. Both these forms of variability need to be considered in deciding the timing of insulin delivery before a meal, to coincide with the glucose peak. Future research should focus on investigating various factors that contribute to the within-person and between-person glycaemic responses. These could include an individual’s mood, hormone levels, exercise levels, unique microbiome, the impact of fat and protein on BGL, pre-meal glucose levels, and gastric emptying rates. Through an increased understanding of the impact these factors have on the glycaemic response, and how these factors interact, a more tailored, adaptive, and progressive approach to insulin dosing could be developed to improve overall glycaemic control.

## Figures and Tables

**Table 1:** Macronutrient composition of the standard breakfast provided to participants during the controlled period.

Food Item	Quantity	Carbohydrate (g)	Fat (g)	Protein (g)	Energy (kJ)
Sanitarium Weet-Bix	30 g (two biscuits)	22.1	0.4	4.1	492
Devondale Semi Skim Long Life Milk	150 mL	7.8	3	4.8	325.5
Western Star Butter	4 g	˂1	3.2	˂1	120
Tip Top the One Wholemeal Bread	1 slice	13.7	1.2	3.6	361.5
**TOTAL**	NA	43.6	7.8	12.5	1299

Abbreviation: not applicable (NA).

**Table 2:** Demographic characteristics (*n* = 30).

*n*	30
Females (*n*, %)	16 (53)
Age (y, mean, SD)	10.5 (1.9)
Diabetes Duration (y, mean, SD)	3.4 (2.7)
HbA1c (mean, (SD) mmol/mol)	7.5 (0.7)
BMI (mean (SD), kg/m^2^)	18.8 (2.6)
BMI z-score	0.3 (0.9)
Insulin Regimen	
CSII (*n*, %)	26 (86.7)
MDI (*n*, %)	4 (13.3)

Abbreviations: years (y); standard deviation (SD); Glycated haemoglobin A1c (HbA1c); Body Mass Index (BMI); Continuous subcutaneous insulin (CSII); Multiple daily injections (MDI).

**Table 3:** Descriptive statistics of the TTP in each condition and mixed model output depicting the inter- and intra-individual variance components and Intra Class Correlation Coefficient (ICC).

	Controlled	Free-Living	
	Breakfast (CB)	Breakfast (FLB)	Dinner (FLD)
Summary			
*n*	26	30	29
Mean study days (SD) for each participant	5 (1)	12 (2)	10 (2)
Valid CGM traces (*n*)	126	350	394
Mean pre-prandial BGL (mmol/L (SD)) ^#^	6.5 (1.2)	7.7 (3.1)	9.4 (4.2)
TTP (minutes)			
Mean (SD)	104.5 (26.6)	93.2 (19.9)	79.5 (22.3)
Range	58.4–161	56.2–131.8	42.1–134.8
Unconditional Mixed Model			
*n*	22	30	29
TTP (minutes)			
Inter-individual SD *	18.5 (14.2–26.4)	14.1 (11.3–19.0)	5.7 (4.5–7.7)
Intra-individual SD *	38.9 (29.9–55.6)	49.6 (39.5–66.7)	64.5 (51.2–87.2)
ICC	0.184	0.075	0.008

Abbreviations: control breakfast (CB); free-living breakfast (FLB); free-living dinner (FLD); blood glucose level (BGL); time to peak (TTP); standard deviation (SD); Intra Class Correlation Coefficient (ICC). ^#^ CB: finger prick BGL; FLB and FLD: sensor BGL. *Data is presented as standard deviation (SD) (95% Confidence Interval).

**Table 4:** Linear mixed model displaying the parameter, 95% confidence interval (CI), and p value of multiple covariates on the time to peak glycaemic response during each study period.

	Controlled			Free-Living					
	Breakfast (CB)			Breakfast (FLB)			Dinner (FLD)		
	Parameter	95% CI	*p*	Parameter	95% CI	*p*	Parameter	95% CI	*p*
BG before	−3.6	−10.7, 3.6	0.33	NA	NA	NA	NA	NA	NA
SG before	NA	NA	NA	−1.2	−2.9, 0.6	0.19	−7.0	−8.8, −5.3	<0.001
Age	1.7	−6.0, 9.6	0.66	0.1	−4.9, 5.2	0.95	1.7	−3.5, 6.8	0.53
Sex	4.0	−26.8, 34.9	0.8	−5.1	−25.5, 15.3	0.63	−1.7	−21.7, 18.2	0.87
BMIz	−13.2	−31.7, 5.3	0.18	−1.0	−13.6, 11.4	0.87	1.2	−10.8, 13.4	0.83
HbA1c	0.6	−19.0, 20.2	0.95	1.9	−10.3, 14.1	0.76	9.9	−2.0, 21.9	0.11
Pre bolus time	NA	NA	NA	0.1	−0.4, 0.6	0.67	−0.2	−0.7, 0.4	0.57

Abbreviation: control breakfast (CB); free-living breakfast (FLB); free-living dinner (FLD); Blood glucose (BG); Sensor glucose (SG); 95% Confidence Interval (95% CI); not applicable (NA).
